# Exploring bleeding in oral anticoagulant users: assessing incidence by indications and risk factors in the entire nationwide cohort

**DOI:** 10.3389/fphar.2024.1399955

**Published:** 2024-09-19

**Authors:** Jonghyun Jeong, Kyu-Nam Heo, Suhyun Lee, Young-Mi Ah, Sangil Min, Ji Min Han, Ju-Yeun Lee

**Affiliations:** ^1^ College of Pharmacy, Seoul National University, Seoul, Republic of Korea; ^2^ College of Pharmacy, Yeungnam University, Gyeongsangbuk-do, Republic of Korea; ^3^ Department of Surgery, Seoul National University Hospital, Seoul, Republic of Korea; ^4^ College of Pharmacy, Chungbuk National University, Cheongju, Republic of Korea

**Keywords:** oral anticoagulants, bleeding, direct oral anticoagulants, warfarin, nonvalvular atrial fibrillation, venous thromboembolism, ischemic stroke

## Abstract

**Background:**

Oral anticoagulants (OACs) are essential for the prevention and treatment of thromboembolic disorders, but bleeding, a major complication, can have a fatal impact on the patient’s treatment.

**Objectives:**

We aimed to estimate the nationwide, real-world incidence rate of bleeding in patients taking OACs and confirm the incidence by indications and risk factors.

**Methods:**

This cross-sectional study identified OAC users from April 1 to December 31, in both 2019 and 2020, using the HIRA-NPS database. The primary outcome variables were the incidence rate of major bleeding events during OAC treatment and within 30 days of treatment discontinuation. We estimated the adjusted incidence rate ratio (aIRR) in subpopulations.

**Results:**

Among 18,822 OAC users, the incidence rate of major bleeding was 27.9 (95% CI: 24.6–31.5) per 1,000 person-years. The incidence rate of major bleeding was higher in patients with a bleeding history, with an aIRR of 11.51; those at high bleeding risk (HAS-BLED score ≥3), with an aIRR of 1.51; those with high CCI scores ≥3, with an aIRR of 1.88; and those with liver disease, with an aIRR of 1.41. For indications, compared to patients with nonvalvular atrial fibrillation (NVAF), the aIRR of major bleeding was significantly higher at an aIRR of 2.35 in patients undergoing VTE treatment. Patients with ischemic stroke showed a higher incidence of major bleeding with an aIRR of 2.13 than NVAF patients. The aIRR of major bleeding in the oral anticoagulant group, compared to the matched control group, was 2.25 (95% CI: 1.93–2.63).

**Conclusion:**

These findings may be useful for implementing strategies to improve the evaluation and management of anticoagulation-related bleeding.

## Graphical Abstract

**Figure:**
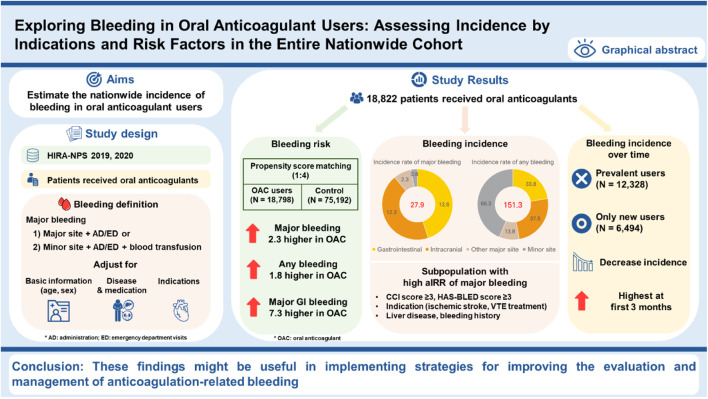

## Introduction

Oral anticoagulants (OACs) are central for the prevention and treatment of numerous thromboembolic disorders. Since their approval by the European Medicines Agency in 2008, direct oral anticoagulants (DOACs), including rivaroxaban, dabigatran, apixaban, and edoxaban, have replaced the long-standing standard therapy of warfarin, an anticoagulant, with vitamin K antagonists (VKA) over the past decade (Franco Moreno et al., 2018). In the United States, the proportion of patients with atrial fibrillation who were prescribed DOACs has increased from 2011 to 2020. Since 2017, the use of DOACs has exceeded that of warfarin (Navar et al., 2022). In Korea, the first DOAC was introduced in 2009, and the reimbursement coverage expanded in 2013 (Son, 2020).

The prevention of stroke and systemic embolism in nonvalvular atrial fibrillation (NVAF), as well as the treatment and prevention of deep vein thrombosis and pulmonary embolism, are commonly approved indications for all oral anticoagulants, except edoxaban, which has not been approved for the postoperative prevention of venous thromboembolism (VTE) after total hip replacement or knee replacement surgery. Unique indications include warfarin for the prevention of valve thrombosis and systemic embolization in patients who received prosthetic heart valves, and rivaroxaban was used for the prevention of major cardiovascular events in patients with chronic coronary artery disease or peripheral artery disease in combination with aspirin (Chen et al., 2020).

Oral anticoagulants have a narrow therapeutic index, and over- or under-treatment can result in significant patient harm, including life-threatening bleeding and stroke. In real practice, they have been consistently identified as the most frequently implicated drug classes in drug-related harm across healthcare settings. Therefore, they have been classified as high-alert or high-risk medications by the Institute of Safe Medication Practices in the United States and many other countries (U.S. Department of Health and Human Services, 2014).

Bleeding is the most common complication associated with OAC use. This can have serious and potentially fatal consequences, leading to the discontinuation of anticoagulation therapy and subsequently increasing the risk of stroke or VTE. The incidence of bleeding varies depending on the type of anticoagulant agent, dosage, indications, treatment duration, concomitant drug use, and the patient population (Ansell, 2012). The risk of bleeding associated with DOACs has been assessed in comparison with warfarin in clinical trials, but those outside clinical trials and in vulnerable populations might differ and need to be investigated.

Previous observational studies have reported the incidence and risk factors of bleeding in specific populations, such as those with atrial fibrillation, older adults with renal impairment, or individuals taking a specific anticoagulant (Fawzy et al., 2019). A propensity score-matched retrospective cohort study reported the incidence rate of first-occurred major bleeding as 2.5 per 100 person-years (PY) for apixaban and 3.7 per 100 PY in patients for warfarin with NVAF (Kohsaka et al., 2018). An observational study in Korea reported that DOACs, including apixaban, dabigatran, and rivaroxaban, were associated with a higher incidence of bleeding than warfarin in patients with NVAF (Bang et al., 2020). A patient-level network meta-analysis reported that patients with AF who received DOACs had a lower incidence of intracranial bleeding than those who received warfarin; however, the incidence of gastrointestinal bleeding was higher (Carnicelli et al., 2022).

Estimating and characterizing the national-level burden of anticoagulant-related harm (hemorrhage) in a real-world setting must be prioritized to evaluate the impact of national action plans to ensure patient safety and medication-related harm prevention efforts. Studies on the nationwide incidence of bleeding in the entire population are limited. Therefore, this study aimed to estimate the nationwide real-world incidence of bleeding complications in patients receiving oral anticoagulants and confirm the incidence by indications and risk factors.

## Materials and methods

### Data source

We used the annual National Patient Sample (NPS) from the Health Insurance Review and Assessment Service (HIRA). The National Health Insurance (NHI) system in South Korea is a universal healthcare program that covers most of the population. It operates on a fee-for-service basis, where healthcare providers are reimbursed for each service rendered. The NHI database primarily comprises claims data submitted by healthcare providers to receive reimbursement for medical services and prescriptions, potentially leading to gaps in data, particularly for non-reimbursed treatments or over-the-counter medications. Despite these limitations, the NHI system offers comprehensive coverage of the Korean population, providing valuable insights into healthcare utilization across diverse demographic groups. Some non-covered drugs may not be included, but most drugs of interest are covered. HIRA reviewed claims, and those for which reimbursement has been completed were stored in the data warehouse. HIRA-NPS was an annual 3% dataset of the national population, stratified by sex and age. The dataset contains comprehensive information such as 1) patient information such as age, sex, and type of insurance; 2) prescription information such as diagnosis and drug codes; and 3) medical practice information such as medical act, number of days of care, and healthcare facility (Kim et al., 2017). This study was approved by the Seoul National University Institutional Review Board (IRB No. E2311/004-005).

### Study population

In this cross-sectional study, we identified patients who were prescribed oral anticoagulants at least once from April 1 to December 31 in both 2019 and 2020. We constructed an oral anticoagulation treatment episode comprising consecutive prescriptions, allowing the gap between any oral anticoagulant prescription to be less than 30 days (Sinyavskaya et al., 2022; Gopalakrishnan et al., 2019). If a different type of anticoagulant was prescribed, previous oral anticoagulant episodes were immediately discontinued, and a new oral anticoagulant episode was initiated in the same patient. Indications for oral anticoagulants were categorized as NVAF, ischemic stroke, VTE, VTE prophylaxis after hip or knee replacement, and valvular heart disease, including valve replacement. Indications were first identified as cases in which the corresponding the 10th revision of the International Classification of Diseases (ICD-10) code was present as the main diagnosis within 3 months before the start of oral anticoagulant administration. Cases in which indications could not be identified using the main diagnosis code were identified using the secondary diagnosis code. If multiple indications were present within 3 months, the last indication was used. Hip/knee replacement was identified using procedural codes (Supplementary Table S1) (Pan et al., 2017).

### Outcome and other variables

The primary outcome variables were the incidence rates of major bleeding events and any bleeding events that occurred during oral anticoagulant treatment and within 30 days of the end of treatment. We classified bleeding sites into four categories corresponding to the ICD-10 codes in the primary diagnosis code: 1) gastrointestinal, 2) intracranial, 3) other major sites (retinal, respiratory, hemarthrosis, and hemothorax), and 4) minor sites (urogenital, nasal, and intraocular). Major bleeding events were defined as hospitalization with a primary diagnosis of bleeding at major sites (gastrointestinal, intracranial, and other major sites) or minor sites requiring blood transfusion. Any bleeding event was defined as the presence of claims with primary diagnosis codes corresponding to bleeding events, regardless of hospitalization or blood transfusion (Supplementary Table S2) (Yun et al., 2019). However, we excluded bleeding events that occurred within 30 days of the previous event, as these events were considered part of the previous bleeding episode. We excluded the bleeding from major trauma with the presence of trauma-related codes. Bleeding events were observed until the end of the observation period, the occurrence of death, or December 31 of each year, whichever occurred first.

Baseline comorbid diseases (hypertension, renal disease, liver disease, peptic ulcer disease, and alcohol abuse) and Charlson Comorbidity Index (CCI) scores were detected with claims during 3 months based on ICD-10 codes. Concomitant use of antiplatelet agents or nonsteroidal anti-inflammatory drugs was also identified.

Baseline bleeding risk was assessed using the HAS-BLED score, assuming a labile INR as a score of 0 because INR information was not included in the claims data. We classified the bleeding risk as low (HAS-BLED score <3) and high (HAS-BLED score ≥3). A history of bleeding was defined as any bleeding occurring within the 3-month observation period (Supplementary Table S3).

### Statistical analysis

Descriptive analysis was used to present the baseline characteristics. The length of medication exposure per patient was calculated, and the summed exposure was presented in patient-years. The incidence rate of outcome events in the total and subgroups was calculated as the number of total events divided by the total exposure (expressed as per 1,000 patient-year), and their 95% confidence intervals (CIs) were calculated.

We estimated the adjusted incidence rate ratio (aIRR) for the subpopulation according to age, sex, CCI score, baseline bleeding risk, indications, hypertension, renal disease, liver disease, peptic ulcer disease, alcohol abuse, history of bleeding, and type of oral anticoagulant. This was done after adjusting for age, sex, CCI score, comorbid disease, concomitant medications, indications, and history of bleeding.

To estimate the relative risk of bleeding complications compared with anticoagulant non-users, a control group was formed after a 1:4 propensity score matching for age, sex, CCI score, comorbid diseases mentioned above, use of concomitant medications, and history of bleeding among patients who were not prescribed oral anticoagulants. The control group was observed for the same period as the patients matched for OAC use, and the aIRR described above was calculated.

To analyze trends in bleeding events over time, we constructed a new user cohort that included only patients who initiated oral anticoagulant therapy for the first time after April 1 without a prescription history during the prior 3 months.

For the *post hoc* analysis of bleeding incidence based on the entire oral anticoagulant users, the bleeding incidence was estimated for each indication. The aIRR for bleeding was adjusted by age, sex, CCI score, comorbidities, concomitant medications, and history of bleeding.

In cases where data were missing at random, the missing data were removed. SAS version 9.4 (SAS Institute) and RStudio 1.4.1717 were used for all statistical analyses.

## Results

### Patient characteristics

**FIGURE 1:**
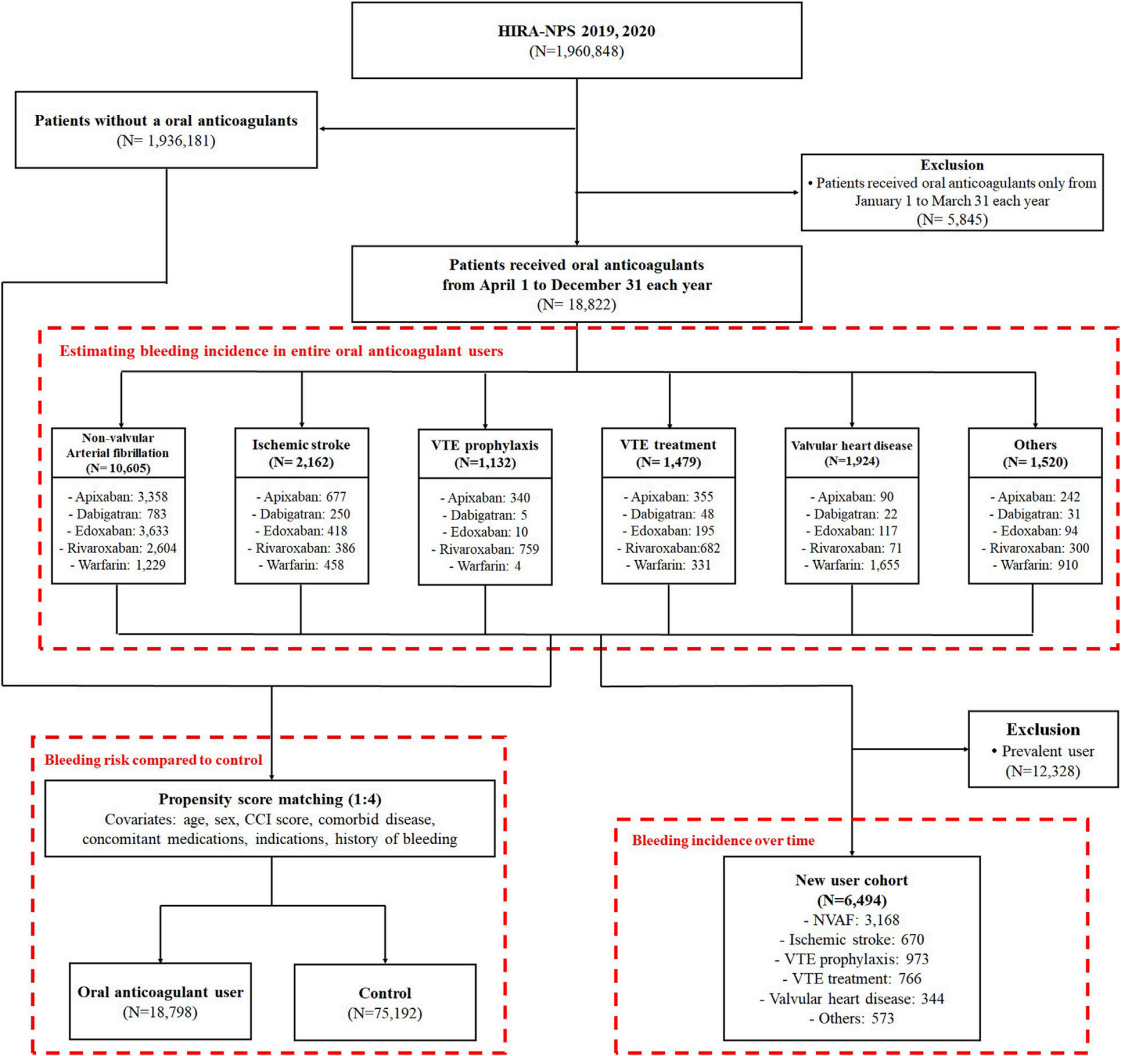
Patient selection process; VTE, venous thromboembolism; NVAF, non-valvular atrial fibrillation.

Among the 1,960,848 patients in the HIRA-NPS in 2019 and 2020, 18,822 patients (1.0%) received oral anticoagulants during the study period (Figure 1). The median follow-up time for patients using oral anticoagulants was 229 days (interquartile range (IQR), 69–275 days), and the total follow-up period was 9,292 person-years. Older adults aged 65–79 years comprised 49.3% of the oral anticoagulant users, while those aged 80 years or older comprised 24.0%. Male sex comprised 52.9% of the users; patients with a CCI score of 3 or more comprised 40.4%, and 46.6% were at high risk of bleeding (HAS-BLED ≥3). The most common indications for OAC use were NVAF at 56.0%, ischemic stroke at 11.4%, valvular heart disease at 10.2%, VTE treatment at 7.8%, and VTE prophylaxis at 6.0%. Among the oral anticoagulants used, apixaban was the most frequently prescribed, accounting for 29.8% (Table 1).

**TABLE 1:** Baseline characteristics of patients with oral anticoagulant.

Characteristics	Oral anticoagulant user, N (%)
Total (N)	18,822
Person-years (years)	9,292
Male	9,955 (52.9)
Age, years	
0–19	57 (0.3)
20–64	4,972 (26.4)
65–79	9,279 (49.3)
80+	4,514 (24.0)
CCI score, mean (STD)	2.43 (1.94)
0	2,478 (13.2)
1–2	8,731 (46.4)
3+	7,613 (40.4)
Indication	
Nonvalvular atrial fibrillation	10,605 (56.0)
Ischemic stroke	2,162 (11.4)
VTE prophylaxis	1,132 (6.0)
VTE treatment	1,479 (7.8)
Valvular heart disease	1,924 (10.2)
Others	1,520 (8.0)
Comorbid condition	
Hypertension	11,754 (62.4)
Peptic ulcer disease	2,514 (13.4)
Liver disease	3,157 (16.8)
Renal disease	1,251 (6.6)
Alcohol abuse	230 (1.2)
HAS-BLED, mean (STD)	2.39 (1.19)
0–2	10,057 (53.4)
3+	8,765 (46.6)
Bleeding history	1,492 (7.9)
Concomitant NSAID or antiplatelets	10,365 (55.1)
Type of oral anticoagulant	
Apixaban	5,602 (29.8)
Dabigatran	1,139 (6.1)
Edoxaban	4,467 (23.7)
Rivaroxaban	4,802 (25.5)
Warfarin	4,587 (24.4)

### Bleeding incidence in entire oral anticoagulant users

The incidence rate of major bleeding in the overall population using oral anticoagulants was 27.9 (95% CI: 24.6–31.5) per 1000 PY, with gastrointestinal bleeding being the most common type at 12.6 per 1000 PY (95% CI: 10.4–15.1). The incidence rate of any bleeding was 151.3 (95% CI: 143.5–159.4) per 1000 PY, with minor site bleeding being most common at 66.3 per 1000 PY (95% CI: 61.2–71.7) (Table 2).

**TABLE 2:** Incidence rate of bleeding.

Bleeding outcome	Oral anticoagulant user	
	Events (N)	Incidence rate (95% CI)
Major bleeding	259	27.9 (24.6–31.5)
Gastrointestinal	117	12.6 (10.4–15.1)
Intracranial	114	12.3 (10.1–14.7)
Other major site^a^	21	2.3 (1.4–3.5)
Minor site	7	0.8 (0.3–1.6)
Any bleeding	1,406	151.3 (143.5–159.4)
Gastrointestinal	314	33.8 (30.2–37.7)
Intracranial	348	37.5 (33.6–41.6)
Other major site	128	13.8 (11.5–16.4)
Minor site	616	66.3 (61.2–71.7)

^a^ Other major site: retinal, respiratory, hemarthrosis, hemothorax.

### Comparison by indications and risk factors

**FIGURE 2:**
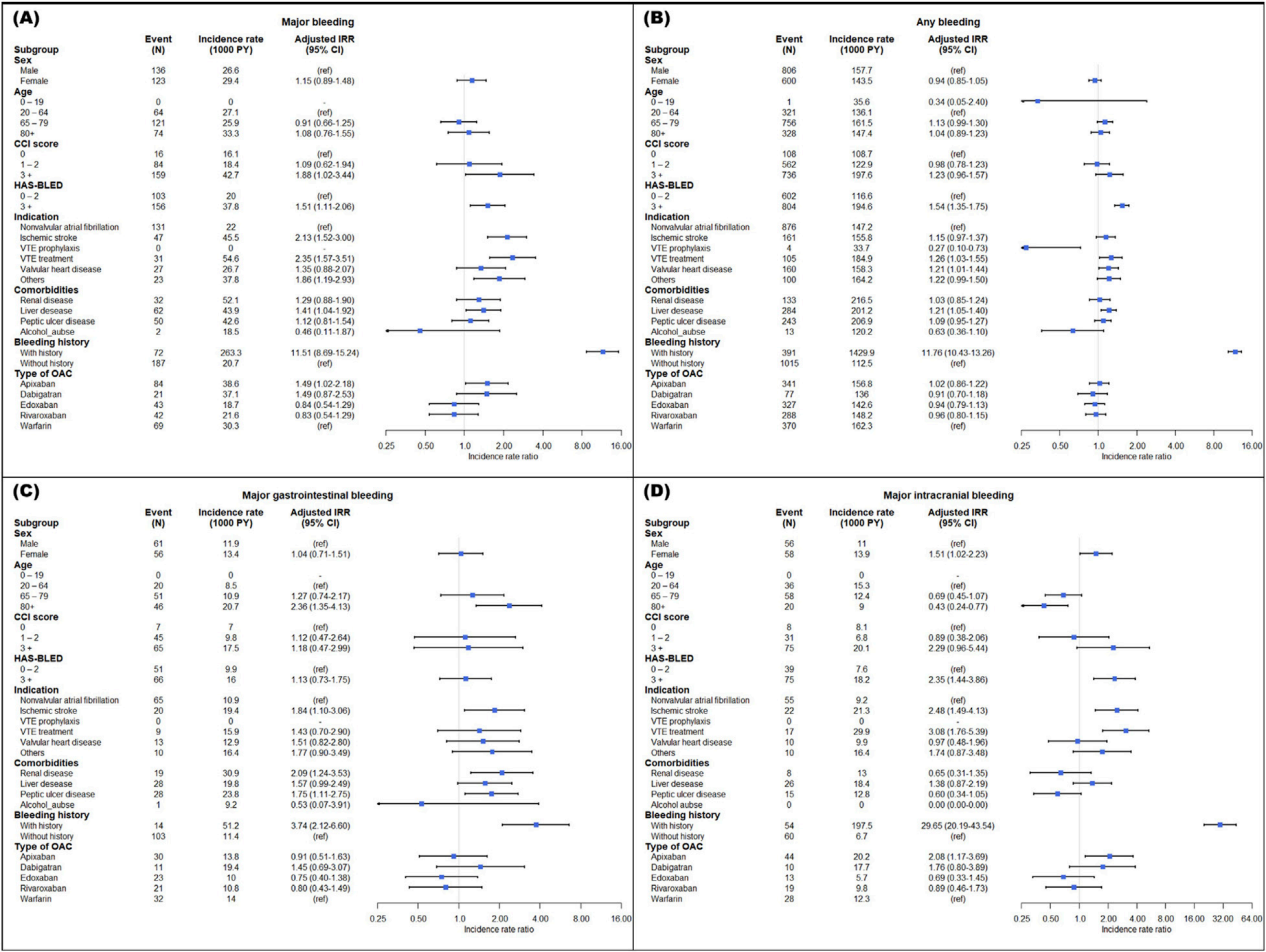
Adjusted incidence rate ratio of major bleeding **(A)**, any bleeding **(B)**, gastrointestinal bleeding **(C)**, and intracranial bleeding **(D)** in subpopulation. IRR, incidence rate ratio; CI, confidence interval; VTE, venous thromboembolism; OAC, oral anticoagulant.

A bleeding history, high bleeding risks with a HAS-BLED score ≥3, a high CCI score of 3 or more, and comorbidities of liver disease were identified as risk factors associated with an increased incidence of major bleeding. The incidence rate of bleeding was higher in patients with a bleeding history (major bleeding, aIRR at 11.51 and 95% CI: 8.69–15.24; any bleeding, aIRR at 11.76 and 95% CI: 10.43–13.26). As noted, patients with a high bleeding risk (HAS-BLED ≥3) had aIRR of 1.51 and 1.54, respectively, higher incidences of major and any bleeding. A high CCI score ≥3 was associated with increased major bleeding with an aIRR of 1.88 (95% CI: 1.02–3.44). Patients with liver disease had a higher incidence of major bleeding and any bleeding than those without liver disease (aIRR 1.41; 95% CI: 1.04–1.92) (Figure 2A).

For indications, in patients undergoing VTE treatment compared to those with NVAF, the aIRRs for major bleeding and any bleeding were significantly higher, recorded at 2.35 (95% CI: 1.57–3.51) and 1.26 (95% CI: 1.03–1.55), respectively. Major bleeding incidence was also greater in patients with ischemic stroke than in those with NVAF, with an aIRR of 2.13 (95% CI: 1.52–3.00). The aIRR for any bleeding increased in patients with valvular heart disease, whereas it decreased in those receiving VTE prophylaxis. Notably, the rate of major bleeding in apixaban users was higher than in warfarin users, with an aIRR of 1.49 (95% CI: 1.02–2.18), as illustrated in Figures 2A,B.

In major gastrointestinal bleeding, patients aged 80 years and older with a high bleeding risk, ischemic stroke, renal disease, peptic ulcer disease, and a history of bleeding showed a significantly high incidence (Figure 2C). Patients aged 80 and older had a low incidence rate compared to those aged 20–64 in intracranial bleeding. Additionally, the aIRRs for major intracranial bleeding were higher in female patients with a high risk of bleeding, ischemic stroke, VTE treatment, a history of bleeding, and apixaban use (Figure 2D).

### Bleeding risk compared to control

After propensity score matching, 18,798 patients using OACs and 75,192 controls were included (Supplementary Table S4). The total follow-up period was 9,281 person-years in the oral anticoagulant user group and 37,126 person-years in the control group. The aIRRs of major bleeding and any bleeding in the oral anticoagulant group compared to the matched control group were 2.25 (95% CI: 1.93–2.63) and 1.78 (95% CI: 1.67–1.90), respectively. The aIRR of the major bleeding of minor sites showed the highest at 26.45 (95% CI: 3.25–215.28), followed by major gastrointestinal bleeding at 7.32 (95% CI: 5.38–9.97) (Table 3).

**TABLE 3:** Incidence rate of bleeding after propensity score matching.

Bleeding outcome	Oral anticoagulant user		Control		Adjusted IRR^a^ (95% CI)
	Events (N)	Incidence rate (95% CI)	Events (N)	Incidence rate (95% CI)	
Major bleeding	256	27.7 (24.3–31.2)	441	11.9 (10.8–13.0)	2.25 (1.93–2.63)
Gastrointestinal	115	12.4 (10.2–14.9)	62	1.7 (1.3–2.1)	7.32 (5.38–9.97)
Intracranial	113	12.2 (10.0–14.6)	341	9.2 (8.2–10.2)	1.30 (1.05–1.61)
Other major site^b^	21	2.3 (1.4–3.5)	37	1.0 (0.7–1.3)	2.22 (1.30–3.79)
Minor site	7	0.8 (0.3–1.6)	1	0.0 (0.0–0.2)	26.45 (3.25–215.28)
Any bleeding	1,393	150.1 (142.3–158.2)	3,029	81.6 (78.7–84.6)	1.78 (1.67–1.90)
Gastrointestinal	310	33.4 (29.8–37.3)	481	13.0 (11.8–14.2)	2.51 (2.17–2.89)
Intracranial	343	37.0 (33.2–41.1)	1,260	33.9 (32.1–35.9)	1.07 (0.95–1.20)
Other major site^b^	126	14.2 (11.3–16.2)	265	7.1 (6.3–8.1)	1.82 (1.47–2.25)
Minor site	614	66.2 (61.0–71.6)	1,023	27.6 (25.9–29.3)	2.37 (2.14–2.62)

^a^ IRR: incidence rate ratio.

^b^ Other major site: retinal, respiratory, hemarthrosis, hemothorax.

### Bleeding incidence over time

The overall incidence of major bleeding decreased over the duration of the anticoagulant use. The first 3 months of anticoagulant use were the highest at 51.1 per 1000 PY (95% CI: 38.9–66.0), followed by an incidence rate of 20.9 per 1000 PY (95% CI: 10.8–36.5) between three and 6 months, and the incidence rate of 15.0 per 1000 PY (95% CI: 4.1–38.5) after 6 months. The incidences of major gastrointestinal bleeding, intracranial bleeding, and other major bleeding sites also decreased over time but not significantly (Supplementary Figure S1).

### Post-hoc analysis

In NVAF patients, the aIRR of major bleeding for edoxaban and rivaroxaban was significantly lower than that of warfarin, with 0.55 (95% CI: 0.32–0.96) and 0.42 (95% CI: 0.23–0.79), respectively. The aIRR for major bleeding for apixaban was lower than that of warfarin (aIRR, 0.71; 95% CI: 0.42–1.22) but not statistically significant (Supplementary Table S5).

## Discussion

In this study, we estimated the nationwide incidence of bleeding in patients receiving oral anticoagulants. The results revealed that the incidence rate of major bleeding was estimated to be 27.9 per 1000 PY, while that for any bleeding was 151.3 per 1000 PY. Although making a direct comparison with previous studies might be challenging due to differences in the type of anticoagulants included, target indication, and study design, the observed incidence rate of major bleeding was similar to or lower than that reported in previous studies. In a retrospective cohort study based on claims data from 314 acute-care hospitals in Japan, the incidence rate of major bleeding with apixaban in patients with NVAF was reported to be 25 per 1000 PY and that with warfarin was reported to be 37 per 1000 PY (Kohsaka et al., 2018). In another nationwide retrospective cohort study in Korea, the crude incidence rate in patients with NVAF who received apixaban was 82.6 per 1000 PY, dabigatran was 78.1 per 1000 PY, rivaroxaban was 94.4 per 1000 PY, and warfarin was 135.3 per 1000 PY (Bang et al., 2020). The observed differences in the incidence rates may be attributed to various factors. Previous studies censored observations when major bleeding occurred, whereas our study repeatedly observed major bleeding during treatment with oral anticoagulants. We included all users of oral anticoagulants regardless of type and indication, whereas previous studies included only new users. It is worth noting that there may be slight differences in defining major bleeding across studies.

We confirmed an increased incidence of bleeding in oral anticoagulant users with previously identified bleeding risk factors. As expected, this study showed that among patients with a high bleeding risk (HAS-BLED score ≥3), the aIRRs for major bleeding, any bleeding, and intracranial bleeding were 1.51, 1.54, and 2.35, respectively, which were similar to previous study results (Gallego et al., 2012). Patients with liver disease had a significantly higher incidence of major bleeding, 1.41 times higher than those without liver disease. When patients experienced any bleeding event within the past 3 months, the incidence rate ratio for major bleeding (11.51), gastrointestinal bleeding (3.74), and intracranial bleeding (29.65) increased significantly. Liver disease and a history of bleeding have been shown to increase the risk of bleeding in previous studies (Hindricks et al., 2021; Benamouzig et al., 2022; Rubboli et al., 2011). Female patients are known to have an increased bleeding risk during anticoagulant therapy, and in this study, the aIRR for major intracranial bleeding was significantly high at 1.51 in female patients (Rubboli et al., 2011).

For indications of anticoagulation, our findings indicate that the incidence of major bleeding was elevated in patients with ischemic stroke and those undergoing VTE treatment compared to those with NVAF. Generally, patients with VTE exhibited a higher bleeding risk relative to those with NVAF, a difference potentially attributable to comorbid conditions and the administration of higher oral anticoagulant dosages in VTE patients (Shoeb and Fang, 2013). Despite adjustments for antiplatelet therapy, ischemic stroke patients maintained a higher major bleeding incidence, suggesting an inherent risk associated with ischemic stroke itself. Hemorrhage transformation is frequently observed in ischemic stroke (Spronk et al., 2021). Our study highlighted a significant increase, with the aIRR for major intracranial bleeding at 2.48. In contrast, patients receiving VTE prophylaxis demonstrated a lower incidence of any bleeding than those with NVAF, likely due to the use of lower dosages.

We observed that the incidence of major intracranial bleeding decreased in patients aged 80 and older, while the incidence of major gastrointestinal bleeding increased in patients aged 80 and older. Previous studies have reported that the hazard ratio of major bleeding increases to 2.9 in elderly patients aged >80 years, with a particularly high risk of gastrointestinal bleeding (Torn et al., 2005; Abraham et al., 2017; Eikelboom et al., 2011; Aisenberg et al., 2018). In this study, the risk of gastrointestinal bleeding was found to be 2.4 times higher in patients aged 80 or older compared to those aged <65 years. However, the incidence of intracranial bleeding decreased in patients aged >65 years compared to that in adult patients, which differs from a previous study. In a previous case-control study, intracranial bleeding showed no significant difference in individuals aged 60–80 years, but significantly higher odds ratio (2.5) were observed in patients aged 85 years and older (Fang et al., 2004). We cannot fully explain this finding, and further investigation is required. However, this difference may be partly attributed to the lower dosage in elderly patients and the more active use of oral anticoagulants in younger patients. Additionally, we were unable to adjust for smoking, uncontrolled hypertension, and disease severity due to claims data and lack of information.

While it is generally known that warfarin, among oral anticoagulants, has a higher incidence of bleeding in NVAF, our study showed that apixaban was associated with a higher incidence of major bleeding than warfarin (Wang et al., 2021; Patel et al., 2011; Connolly et al., 2009). Although we adjusted the major bleeding aIRR with other covariates, including indications, there might have been selection bias because warfarin was mostly prescribed for valvular heart disease, whereas other oral anticoagulants were not prescribed. The incidence of major bleeding according to the type of oral anticoagulant used in the patients is shown in Supplementary Table S5. Compared to warfarin in NVAF patients, the aIRRs of major bleeding of edoxaban and rivaroxaban were significantly lower at 0.55 (95% CI: 0.32–0.96) and 0.42 (95% CI: 0.23–0.79), respectively. The aIRR of apixaban was low but not significant, which is due to the limited warfarin use in patients with NVAF and the short observation period. Currently, warfarin is not recommended for patients with NVAF; therefore, this study included patients who were prescribed warfarin (Kirchhof et al., 2016; Lip et al., 2018; Chiang et al., 2017). Most warfarin users are considered prevalent users who have been taking warfarin for a long time. For these reasons, it seems to have caused the difference in the known bleeding incidence between DOACs and warfarin.

We also observed that the incidence rate of major bleeding was approximately 2.3 times higher in oral anticoagulant users than that in the propensity-matched cohort that did not use oral anticoagulants. The highest increase was observed in minor-site major bleeding, which was 26 times higher, followed by gastrointestinal bleeding, which increased 7.3 times. However, the incidence of intracranial bleeding showed only a slight increase in patients using oral anticoagulants. It is noteworthy that the inclusion of patients with a higher risk of bleeding despite not using anticoagulants might have contributed to these results.

The bleeding incidence during the initial 3 months of starting oral anticoagulant treatment was 51.1 (95% CI: 38.9–66.0), which is significantly higher than the incidence of 20.9 (95% CI: 10.8–36.5) during the 3–6 month period (Supplementary Figure S1). In previous studies, among patients with AF who received warfarin, a significantly higher incidence of major bleeding was observed within the initial 3-month period (Hylek et al., 2007). In a multicenter prospective cohort study, the incidence of bleeding during the first 6 months of oral anticoagulant therapy was significantly higher than that between 6 and 12 months (Bouget et al., 2020). This result indicates that patients with newly initiated oral anticoagulant therapy require closer monitoring.

This study has several limitations. First, the determination of bleeding events relied on the primary diagnoses recorded in the claims data. It is possible that errors or omissions in the diagnosis codes led to an underestimation or overestimation of the incidence rate of bleeding. However, for the definition of major bleeding, we considered hospitalization, transfusion, and the main diagnosis to minimize potential errors associated with diagnostic codes. Second, our analysis was based on 1-year sample claims data, which did not include laboratory values, such as INR. The absence of INR values may have resulted in underestimating the HAS-BLED score. Second, limited access to patient information from previous years restricted our ability to obtain more complete information, potentially leading to inaccuracies in identifying indications and assessing baseline comorbidities. Third, the accuracy and completeness of the dataset may be compromised due to potential coding and data entry errors, leading to possible misclassification and incomplete information. Diagnosis and procedure codes might not always capture the true clinical scenarios. Additionally, the dataset does not include data on over-the-counter medications, which could result in incomplete drug utilization information and a potential underestimation of medication exposure. Furthermore, due to the observational nature of the dataset, there is a risk of selection bias, and the absence of detailed demographic information may limit our ability to control all confounding variables fully. Next, the HIRA-NPS dataset is specific to Korea, which may limit the generalizability of our findings to other populations with different healthcare systems and demographic characteristics. Despite these limitations, we have employed rigorous statistical methods to mitigate the impact of these potential biases and ensure robust data analysis. Lastly, dose adjustment according to renal function, age, and weight was not considered. Our results did not show an increased risk of bleeding with age, which may be due to the lack of considering dose adjustment for age.

## Conclusion

We observed that the incidence rates of major and any bleeding among oral anticoagulant users were 27.9 per 1000 PY and 151.3 per 1000 PY, respectively. These rates were 2.3 times higher than those of non-users. We observed a significant increase in the incidence of major bleeding among patients with a high CCI score ≥3, high bleeding risk (HAS-BLED score ≥3), the indication of ischemic stroke or VTE treatment, liver disease, and a history of bleeding. To the best of our knowledge, this study is the first to estimate the nationwide bleeding incidence in oral anticoagulant users regardless of indications for the first time and compare the risk according to patient characteristics and conditions. These findings may be useful in implementing strategies to improve the evaluation and management of anticoagulation-related bleeding.

## Data Availability

The original contributions presented in the study are included in the article/Supplementary Material; further inquiries can be directed to the corresponding authors.
