# Supplementary material for: Exploring bleeding in oral anticoagulant users: assessing incidence by indications and risk factors in the entire nationwide cohort
Source: Front Pharmacol. 2024 Sep 19;15:1399955. doi: 10.3389/fphar.2024.1399955 (PMC11446751; doi:10.3389/fphar.2024.1399955)
Supplement: Supplementary file 1 [file Table1.docx]

Supplementary Table S1. Potential indication ICD-10 diagnosis code

| **Potential indication** | **ICD-10 diagnosis** | **Medical act code** |
| --- | --- | --- |
| Atrial fibrillation | I48 | - |
| Ischemic stroke | I63 – I64 |  |
| VTE treatment | I26, I80-I82, O082, O223, O871 |  |
| VTE prophylaxis |  | N0711, N0715, N2072, N2712 |
| Valvular heart disease | I05, I08, I34, T820, T826, Z952, Z953, Z954 | O1782, O1792, O1793, O1799 |
