# Supplementary material for: Exploring bleeding in oral anticoagulant users: assessing incidence by indications and risk factors in the entire nationwide cohort
Source: Front Pharmacol. 2024 Sep 19;15:1399955. doi: 10.3389/fphar.2024.1399955 (PMC11446751; doi:10.3389/fphar.2024.1399955)
Supplement: Supplementary file 2 [file Table2.docx]

Supplementary Table S2. Bleeding ICD-10 diagnosis code

| **Bleeding site** | **ICD-10 diagnosis** |
| --- | --- |
| **Major site** | |
| Gastro-intestinal bleeding | I850, K22.11, K22.8, K25.0, K25.2, K25.4, K25.6, K26.0, K26.2, K26.4, K26.6, K27.0, K27.2, K27.4, K27.6, K28.0, K28.2, K28.4, K28.6, K29.0, K31.81, K55.21, K57.01, K57.03, K57.11, K57.13, K57.21, K57.23, K57.31, K57.33, K57.41, K57.43, K57.51, K57.53, K57.81, K57.83, K57.91, K57.93, K62.5, K66.1, K92.0, K92.1, K92.2 |
| Intracranial bleeding | I60.X, I61.X, I62.X, S06.4 |
| Other major site bleeding | D62, H05.2, H35.6, H43.1, M25.0, R04, R04.1, R04.2, R04.8, R04.9, J94.2 |
| **Minor site** | |
| Urogenital bleeding | N50.1, N83.0, R31, R31.0, R31.8 |
| Nasal bleeding | R04.0 |
| Intraocular bleeding | H11.3, H21.0, H31.3, H47.0 |
| Other minor site bleeding | D68.3, E27.4, R58 |
