# Supplementary material for: Exploring bleeding in oral anticoagulant users: assessing incidence by indications and risk factors in the entire nationwide cohort
Source: Front Pharmacol. 2024 Sep 19;15:1399955. doi: 10.3389/fphar.2024.1399955 (PMC11446751; doi:10.3389/fphar.2024.1399955)
Supplement: Supplementary file 3 [file Table5.docx]

Supplementary Table S5. Incidence rate of major bleeding in non-valvular atrial fibrillation patients

| Oral anticoagulants | Major bleeding | | |
| --- | --- | --- | --- |
|  | Events  (N) | Incidence rate  (per 1000 PY) | Adjusted IRR*  (95% CI) |
| Apixaban | 40 | 24.7 | 0.71 (0.42-1.22) |
| Dabigatran | 16 | 40.6 | 1.17 (0.60-2.27) |
| Edoxaban | 34 | 17.5 | 0.55 (0.32-0.96) |
| Rivaroxaban | 20 | 14.6 | 0.42 (0.23-0.79) |
| Warfarin | 21 | 34.4 | (ref) |

* IRR: incidence rate ratio
