# Supplementary material for: Exploring bleeding in oral anticoagulant users: assessing incidence by indications and risk factors in the entire nationwide cohort
Source: Front Pharmacol. 2024 Sep 19;15:1399955. doi: 10.3389/fphar.2024.1399955 (PMC11446751; doi:10.3389/fphar.2024.1399955)
Supplement: Supplementary file 4 [file Table3.docx]

Supplementary Table S3. HAS-BLED score ICD-10 diagnosis code

| **HAS-BLED** | **ICD-10 diagnosis** | **Drugs** |
| --- | --- | --- |
| H, hypertension | I10 | - |
| A, abnormal renal function | N183, N184 | - |
| A, Abnormal liver function | B15, B16, B17, B19, C22, D684, I982, K70, K71, K72, K73, K74, K75, K76, K77, Z944 | - |
| S, stroke | I63, I64, I69 | - |
| B, bleeding |  | - |
| E, elderly (> 65 years) | - |  |
| D, alcohol | E244, F10, G312, G6221, G721, I426, K292, K70, K860, O354, P043, Q860, T510, X45, X65, Y15, Y90, Y91, Z502, Z714, Z721 |  |
| D, drug |  | NSAIDs and antiplatelet agents |
