# Supplementary material for: Exploring bleeding in oral anticoagulant users: assessing incidence by indications and risk factors in the entire nationwide cohort
Source: Front Pharmacol. 2024 Sep 19;15:1399955. doi: 10.3389/fphar.2024.1399955 (PMC11446751; doi:10.3389/fphar.2024.1399955)
Supplement: Supplementary file 5 [file Table4.docx]

Supplementary Table S4. Baseline characteristics after propensity score matching

| Characteristics | Oral anticoagulant user,  N (%) | Control,  N (%) | Standard mean difference |
| --- | --- | --- | --- |
| Total (N) | 18,798 | 75,192 |  |
| Person-years (years) | 9,281 | 37,126 |  |
| Male | 9,934 (52.8) | 39,652 (52.7) | 0.002 |
| Age, years |  |  |  |
| 0 – 19 | 57 (0.3) | 222 (0.3) | 0.001 |
| 20 – 64 | 4,972 (26.4) | 19,729 (26.2) | 0.004 |
| 65 – 79 | 9,272 (49.3) | 37,133 (49.4) | -0.001 |
| 80+ | 4,497 (23.9) | 18,108 (24.1) | -0.004 |
| CCI score, mean (STD) | 2.42 (1.92) | 2.41 (1.90) | 0.009 |
| 0 | 2,478 (13.2) | 9,936 (13.2) | -0.001 |
| 1 – 2 | 8,731 (46.4) | 35,006 (46.6) | -0.002 |
| 3 + | 7,589 (40.4) | 30,250 (40.2) | 0.003 |
| Indication |  |  |  |
| Nonvalvular atrial fibrillation | 10,727 (57.1) | - | - |
| Ischemic stroke | 2,013 (10.7) | - | - |
| VTE prophylaxis | 1,117 (5.9) | - | - |
| VTE treatment | 1,512 (8.0) | - | - |
| Valvular heart disease | 1,902 (10.1) | - | - |
| Others | 1,510 (8.0) | - | - |
| Comorbid condition |  |  |  |
| Hypertension | 11,731 (62.4) | 47,190 (62.8) | -0.007 |
| Peptic ulcer disease | 2,505 (13.3) | 9,840 (13.1) | 0.007 |
| Cancer | 1,528 (8.1) | 5,998 (8.0) | 0.006 |
| Liver disease | 3,143 (16.7) | 12,659 (16.8) | -0.003 |
| Renal disease | 1,235 (6.6) | 4,799 (6.4) | 0.008 |
| Alcohol abuse | 230 (1.2) | 906 (1.2) | 0.002 |
| HAS-BLED, mean (STD) | 2.31 (1.16) | 2.24 (1.15) | 0.060 |
| 0 – 2 | 10,466 (55.7) | 44,104 (58.7) | -0.060 |
| 3 + | 8,332 (44.3) | 31,088 (41.3) | 0.060 |
| Bleeding history | 538 (2.9) | 2,048 (2.7) | 0.008 |
| Concomitant NSAID or antiplatelets | 10,332 (55.0) | 41,551 (55.3) | -0.006 |
